# Supplementary material for: Accurately modeling biased random walks on weighted networks using node2vec+
Source: Bioinformatics. 2023 Jan 23;39(1):btad047. doi: 10.1093/bioinformatics/btad047 (PMC9891245; doi:10.1093/bioinformatics/btad047)
Supplement: btad047_Supplementary_Data [file btad047_supplementary_data.pdf]

Supplemental Information for Accurately modeling biased random walks on weighted networks using *node2vec+*

Hierarchical cluster graphs construction details

The hierarchical cluster graphs are constructed by first sampling in a representation space constructed based on the corresponding tree structure and then applying an RBF kernel.

Tree construction

We first construct the cluster centroids using a tree structure. A *perfect binary tree* is a binary tree where all nodes except the leaf nodes have two children, and all leaf nodes have the same level. This definition can be generalized to *perfect K-trees*, in which all the interior nodes have K number of nodes, for K greater than or equal to one. We denote $T_{K,L}$ as the *perfect K-tree* with maximum level L. Figure S1 shows the example of $T_{2,2}$, a *perfect binary tree* (or *perfect 2-tree*) with a maximum level of two.

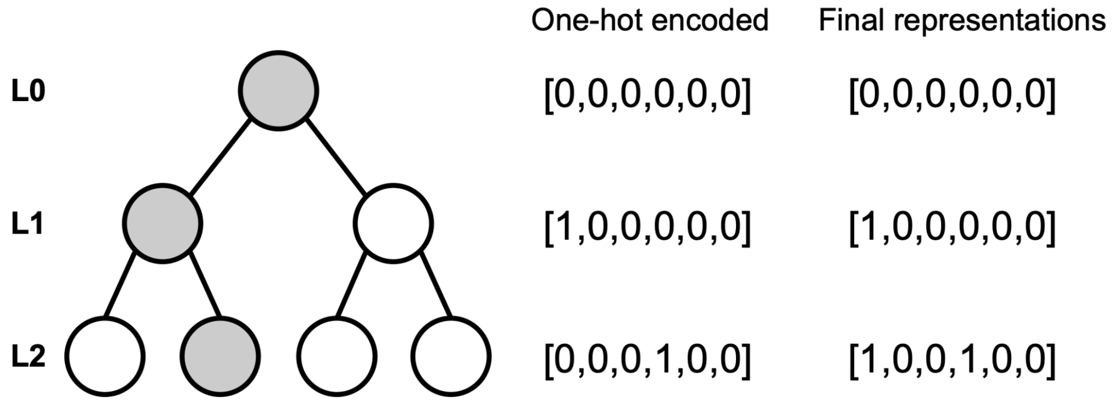

Figure S1. A perfect binary tree with two levels.

Representing nodes in the tree

A straightforward solution to represent the nodes in $T_{K,L}$ is one-hot encoding. For a more compact representation, we leave out the indicator for the root node and represent it as all zeros. Thus, the dimension of the indicator array is equal to the total number of nodes in $T_{K,L}$, excluding the root node, that is,

$$|V(T_{K,L})| - 1 = \sum_{l=1}^{L} K^{l}$$

However, if we use one-hot encoding, then all nodes are equally distanced in the Euclidean space. Instead, we combine the one-hot encoded representations of all the ancestor nodes as the final representation for each node, denoted as μ_i, $i = 1,\ldots, |V(T_{K,L})|$. In this way, all sibling

nodes are equally distanced, with $\sqrt{2}$ times the distance from the parent node. Figure S1 shows the example of both the one-hot encoded and the final representations of the grey nodes. Notice the difference between the final representation and the on-hot encoded representation of the grey leaf node.

Hierarchical clusters

We draw data points x from a Gaussian distribution around each node in the $T_{K,L}$ tree:

$$x \sim N(\mu_i, \sigma),\ i = 1,..., |V(T_{K,L})|$$

In the case of K3L2, the data points are drawn using $T_{3,2}$. The parameter σ, which controls the noisiness of the sampled data points, is set to 0.01 by default. Throughout the study, we fix the number of data points per node in the tree to 30. Finally, we turn the sampled data points into a fully connected weighted graph using the RBF kernel.

Maximal sparsification of K3L2

We apply a global edge threshold to K3L2 by removing all edges below a certain value. Sweeping through [0.01, 0.9], we found that the maximum global edge threshold that preserves the graph's connectivity is about 0.45 (Figure S2). Notice that the edge density drastically reduces by doing so to around 0.1.

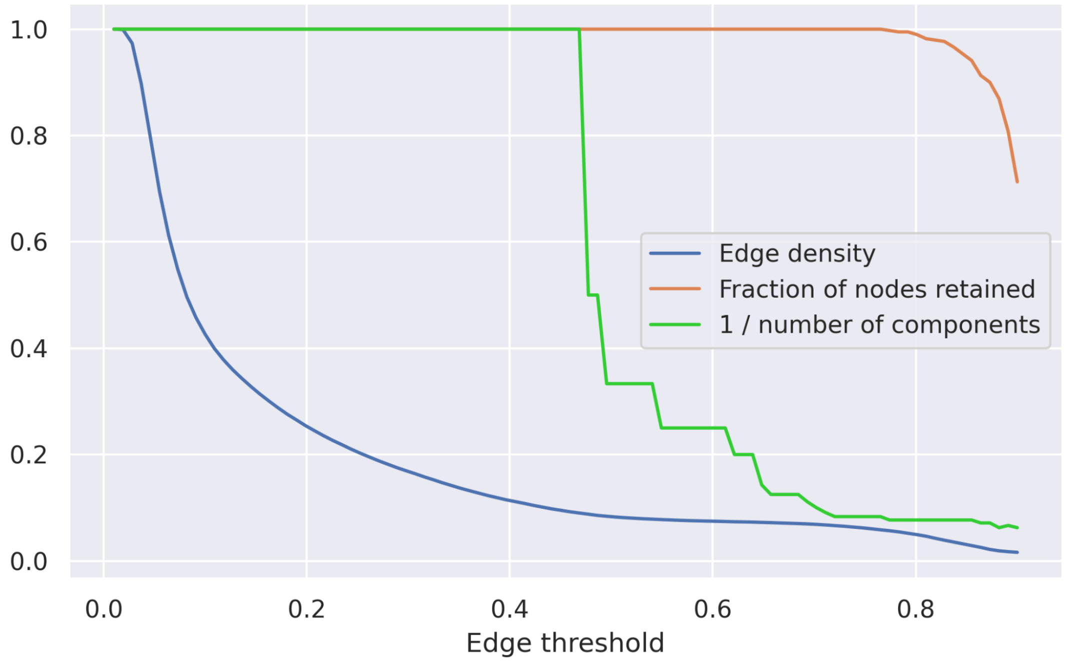

Figure S2. Network statistics of K3L2 as a function of sparsifying edge threshold.

Gene classification datasets

Gene interaction networks

We first downloaded gene interaction networks from STRING (https://string-db.org/) and HumanBase (https://humanbase.flatironinstitute.org/about). The GTEx tissue-specific gene co-expression networks were obtained from the data archive associated with Johnson and Krishnan (2022). To generate a tissue-naive GTEx gene co-expression network, we first used the script provided in https://github.com/krishnanlab/RNAseq_coexpression to generate five tissue-naive networks, each was constructed using 312 randomly selected from the GTEx gene expression dataset. The final GTEx tissue naive network (global) was taken as the average of the five bootstrapped co-expression networks.

Since each type of network uses a different gene identifier, more specifically, STRING uses Ensembl proteins, GTEx networks use Ensembl genes, and HumanBase uses Entrez, we converted all networks to the Entrez gene identifier. The ID conversion was achieved using the MyGeneInfo query service (https://mygene.info/). Finally, we took the largest connected component of each network and discarded other disconnected genes.

Gene annotations

We followed the procedures detailed in GenePlexus (Liu *et al.,* 2022) closely to construct the GOBP and DisGeNET datasets. In brief, we first downloaded the gene ontology and disease ontology from the OBOFoundry (https://obofoundry.org/), along with the gene annotations from http://geneontology.org/ and https://www.disgenet.org/. Then, we propagated the gene annotations along the ontologies and extracted non-redundant representative gene sets of reasonable sizes (GOBP between 50 and 200, DisGeNET between 50 and 600). These gene sets are further filtered in the next step based on the study-bias train/val/test split.

The study-bias split was done by splitting the genes based on the level they have been studied in the past according to the number of PubMed articles associated with them (https://pubmed.ncbi.nlm.nih.gov/download/). More specifically, for each gene set collection (GOBP or DisGeNET), we sort the genes present in the collection based on their PubMed count. The top 60% of genes with the most PubMed count are used for training, the 20% of genes with the least PubMed count are used for testing, and the rest are used for validation. We remove any gene sets whose minimum number of positive examples in any splits is less than ten.

Tissue-specific gene annotations

We downloaded the tissue-specific gene function annotations presented in OhmNet (Zitnik & Leskovec, 2017) from http://snap.stanford.edu/ohmnet/.

Table S1. Gene interaction network statistics.

Network	# Nodes	# Edges	Edge density
STRING	17,352	3,640,737	2.42E-02
HumanBase-global	24,114	290,730,441	1.00E+00
HumanBaseTop-global	24,003	33,937,714	1.18E-01
HumanBase-blood	24,114	290,730,441	1.00E+00
HumanBase-blood_vessel	24,114	290,730,441	1.00E+00
HumanBase-brain	24,114	290,730,441	1.00E+00
HumanBase-heart	24,114	290,730,441	1.00E+00
HumanBase-kidney	24,114	290,730,441	1.00E+00
HumanBase-muscle	24,114	290,730,441	1.00E+00
HumanBaseTop-blood	24,003	29,451,338	1.02E-01
HumanBaseTop-blood_vessel	24,114	55,910,520	1.92E-01
HumanBaseTop-brain	24,003	36,199,871	1.26E-01
HumanBaseTop-heart	24,114	48,614,273	1.67E-01
HumanBaseTop-kidney	24,114	48,599,506	1.67E-01
HumanBaseTop-muscle	24,114	54,492,105	1.87E-01
GTExCoExp-blood	19,809	95,535,108	4.87E-01
GTExCoExp-blood_vessel	19,809	97,085,845	4.95E-01
GTExCoExp-brain	19,809	98,573,534	5.02E-01
GTExCoExp-global	20,116	98,135,412	4.85E-01
GTExCoExp-heart	19,809	97,582,341	4.97E-01
GTExCoExp-kidney	19,809	97,929,900	4.99E-01
GTExCoExp-muscle	19,809	97,240,974	4.96E-01
GTExCoExpTop-blood	19,809	7,884,935	4.02E-02
GTExCoExpTop-blood_vessel	19,809	4,879,744	2.49E-02
GTExCoExpTop-brain	19,809	6,276,541	3.20E-02
GTExCoExpTop-global	20,116	9,959,240	4.92E-02
GTExCoExpTop-heart	19,809	5,067,436	2.58E-02
GTExCoExpTop-kidney	19,809	8,589,096	4.38E-02
GTExCoExpTop-muscle	19,809	5,415,950	2.76E-02

Graph Neural Networks

Basic architecture design

The basic GNN architecture we used consists of three main components: (1) the pre-message-passing (pre-mp) layer that map the initial node features to the hidden dimension, (2) the graph convolution layers, and (3) the post-message-passing (post-mp), or the prediction head, layer that maps the final node embeddings to the prediction values. In addition, the convolution layers have the option to add residual (skipsum) connections. We initialize the linear (pre-/post-mp) layers using Xavier uniform initialization. Finally, to train the GNNs, we use the standard Adam optimizer with a *reduce learning rate on plateau* scheduler, along with dropout and weight decay.

Hyperparameter tuning

We thoroughly tuned the GNN architectures and training settings based on the median of the validation performance, measured by log2 fold change of average precision over the prior, across all datasets, with three repeated runs. More specifically, we separate the tuning into two stages. In the first stage, we tune the architecture parameters (hidden dimension, number of convolution layers, and residual connection) via grid search while fixing the training parameters (learning rate, dropout rate, weight decay) to reasonable settings, as shown in Table S2. Then, in the second stage, we fix the optimally tuned architecture for each convolution type (GCN or GraphSAGE) and tune the training parameters via grid search. The resulting optimal settings[1] for the GNNs are shown in Table S3.

[1] The full analysis can be found in this notebook: https://github.com/krishnanlab/node2vecplus_benchmarks/blob/main/gnn_tuning/hyperparameters_tuning.ipynb

Table S2. Hyperparameters grids and default training settings.

Type	Name	Default	Grid
Architecture	Hidden dimension	-	[16, 32, 64, 128]
Architecture	Number of convolution layers	-	[3, 4, 5]
Architecture	Residual connection	-	[True, False]
Training	Learning rate	1e-3	[1e-4, 1e-3, 1e-2, 1e-1]
Training	Dropout rate	0.1	[0.0, 0.1, 0.3]
Training	Weight decay	1e-5	[1e-4, 1e-5, 1e-6, 1e-7]

Table S3. Optimally tuned settings for GCN and GraphSAGE.

	Hidden dimension	Number of convolution layers	Residual connection	Learning rate	Dropout rate	Weight decay
GCN	128	5	True	1e-2	0.0	1e-7
GraphSAGE	128	5	False	1e-3	0.1	1e-5

Training information

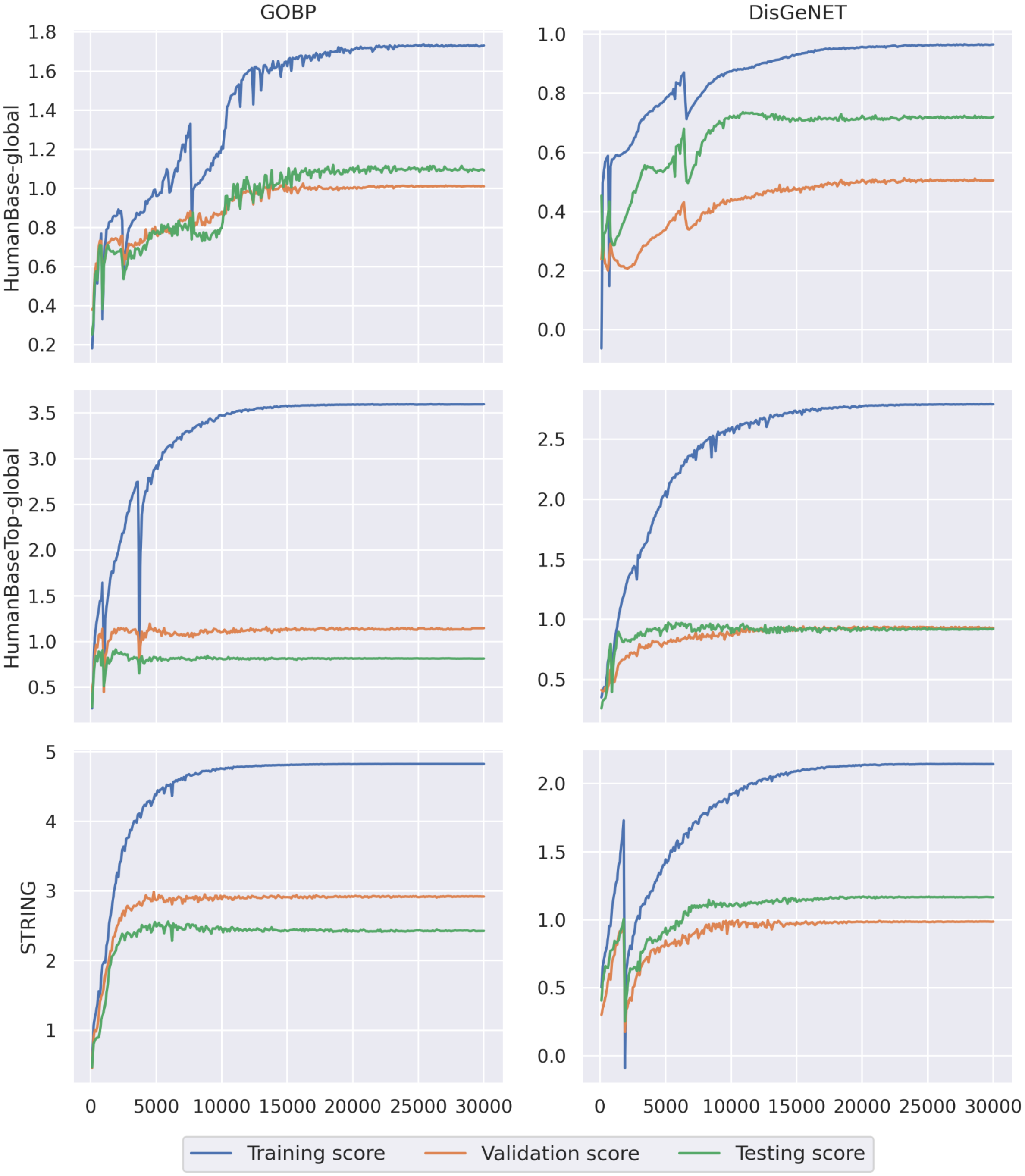

Figure S3. GCN training progress.

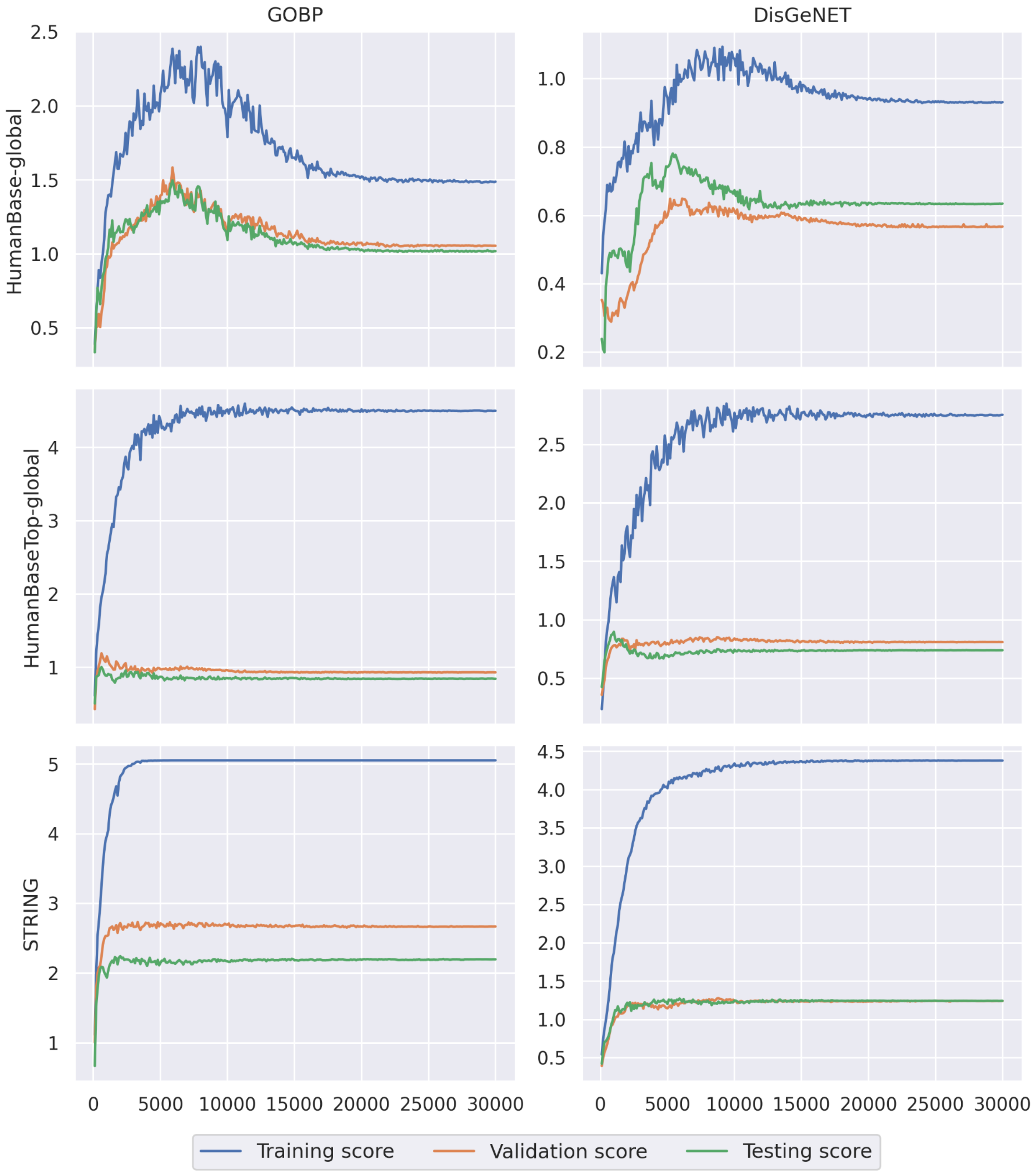

Figure S4. GraphSAGE training progress.

Node2vec++: continuous extension of *node2vec+*

By design of node2vec+, more specifically the noisy-edge term, there is a discontinuity in the bias factor function α. In this section, we first review the discontinuity issue and provide an alternative solution, called *node2vec++*, which continuously extends *node2vec+*. We finally empirically show, using the gene classification tasks, that *node2vec+* performs better than the continuous extension version.

Recall that the bias factor function for *node2vec+* is defined as follows

$\frac{1}{p}$	$if\ v_p = v_n$	(S1.1)
1	$if\ \tilde{w}_\gamma(v_n, v_p) \geq 1$	(S1.2)
$\min\{1, \frac{1}{q}\}$	$if\ \tilde{w}_\gamma(v_n, v_p) < 1\ and\ \tilde{w}_\gamma(v_c, v_n) < 1$	(S1.3)
$\frac{1}{q} + (1 - \frac{1}{q})\tilde{w}_\gamma(v_n, v_p)$	$if\ \tilde{w}_\gamma(v_n, v_p) < 1\ and\ \tilde{w}_\gamma(v_c, v_n) \geq 1$	(S1.4)

Equation S1. Bias factor for *node2vec+*.

Notice that if $\tilde{w}_\gamma(v_n, v_p) < 1$, then as $\tilde{w}_\gamma(v_c, v_n)$ changes from greater than one to less than one, or vice versa, the switching between S1.3 and S1.4 causes a sudden change of the bias factor, so long as q is not 1.

To resolve the discontinuity issue, we make use of the function $y_\beta(z) = \frac{\beta z}{1 + (\beta - 1)z}$ (see Figure S5), which interpolate between the linear function $y_1(z) = z$ and the constant function $y_0(z) = 0$, for $z \in [0, 1)$. Since by design, S1.3 always suppresses the bias factor, we need to modify $y_\beta(z)$ to take z or $1 - z$ as input depending on whether q is greater or less than 1. Thus, we have the following formulation for *node2vec++*

$\frac{1}{p}$	$if\ v_p = v_n$	(S2.1)
1	$if\ \tilde{w}_\gamma(v_n, v_p) \geq 1$	(S2.2)
$\rho * \lvert 1 - \frac{1}{q} \rvert + \min\{1, \frac{1}{q}\}$	$if\ \tilde{w}_\gamma(v_n, v_p) < 1$	(S2.3)

Equation S2. Bias factor for *node2vec++*.

where S2.3 is a reparameterization of ρ from $[0, 1]$ to $[\min\{1, \frac{1}{q}\}, \quad \max\{1, \frac{1}{q}\}]$, and ρ is defined based on the interpolation function $y_{\beta}(z)$ mentioned above as follows

$\frac{\tilde{w}_{\gamma}(v_c, v_n)\tilde{w}_{\gamma}(v_n, v_p)}{1+(\tilde{w}_{\gamma}(v_c, v_n)-1)\tilde{w}_{\gamma}(v_n, v_p)}$	$if\ q \geq 1$	(S3.1)
$\frac{\tilde{w}_{\gamma}(v_c, v_n)(1-\tilde{w}_{\gamma}(v_n, v_p))}{1+(\tilde{w}_{\gamma}(v_c, v_n)-1)(1-\tilde{w}_{\gamma}(v_n, v_p))}$	$if\ q < 1$	(S3.2)

Equation S3. Interpolation function.

The visualization of the bias factor α (z-axis) as a function of $\tilde{w}_{\gamma}(v_c, v_n)$ and $\tilde{w}_{\gamma}(v_p, v_c)$ is shown in Figure S5. In particular, when q is not 1, *node2vec+* (left column) admits discontinuity in the bias factor surface, while *node2vec++* (right column) continuously extends the *node2vec+* bias factor surface. Furthermore, we make the following remarks for *node2vec++*.

1. The bias factor α is continuous with respect to the normalized edge weights $\tilde{w}_{\gamma}(v_c, v_n)$ and $\tilde{w}_{\gamma}(v_n, v_p)$.
2. *Node2vec++* is a continuous extension of *node2vec+* for cases where $\tilde{w}_{\gamma}(v_c, v_n) < 1$ and $\tilde{w}_{\gamma}(v_n, v_p) < 1$, in which case $\alpha_{n2v+} = \lim_{\tilde{w}_{\gamma}(v_c, v_n) -> 0} \alpha_{n2v++}$.
3. *Node2vec++* reduces to *node2vec* in the case of unweighted graph, so does *node2vec+*. This can be seen by first realizing that, in the usual case, e.g., $\gamma = 0$, $\tilde{w}_{\gamma}$ can either be zero or one. Then, plugging in the two cases into S1 and S2 results in only three possible outcomes of $\frac{1}{p}$, 1, and $\frac{1}{q}$, appearing in the corresponding situations required by *node2vec*.

We conducted the gene classification experiments discussed in section 3.2.4 using *node2vec++* and compared the performance against *node2vec+*. It can be seen from Figure S7 that *node2vec+* generally outperformed *node2vec++*. We hypothesized that the performance differences are due to a stronger denoising effect of *node2vec+* as it squashes the bias factor to $\min\{1, \frac{1}{q}\}$ instead of gradually reducing the bias factor as in *node2vec++*. One possible

solution to increase the performance of *node2vec++* is to introduce an additional hyperparameter that controls how “fast” the reduction occurs, e.g., by raising S2.3 to some power or multiplying it with some value. We decided to use *node2vec+* in the main paper due to its simplicity and good empirical performance.

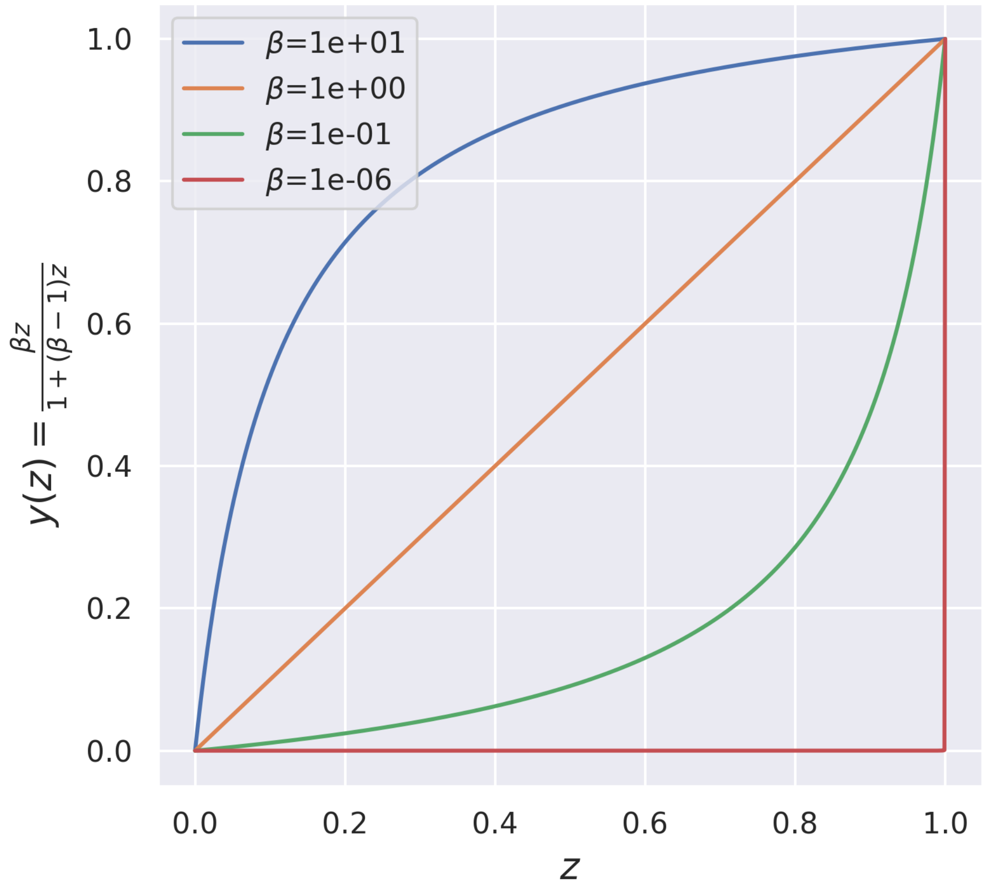

Figure S5. Visualization of the interpolation function used in node2vec++.

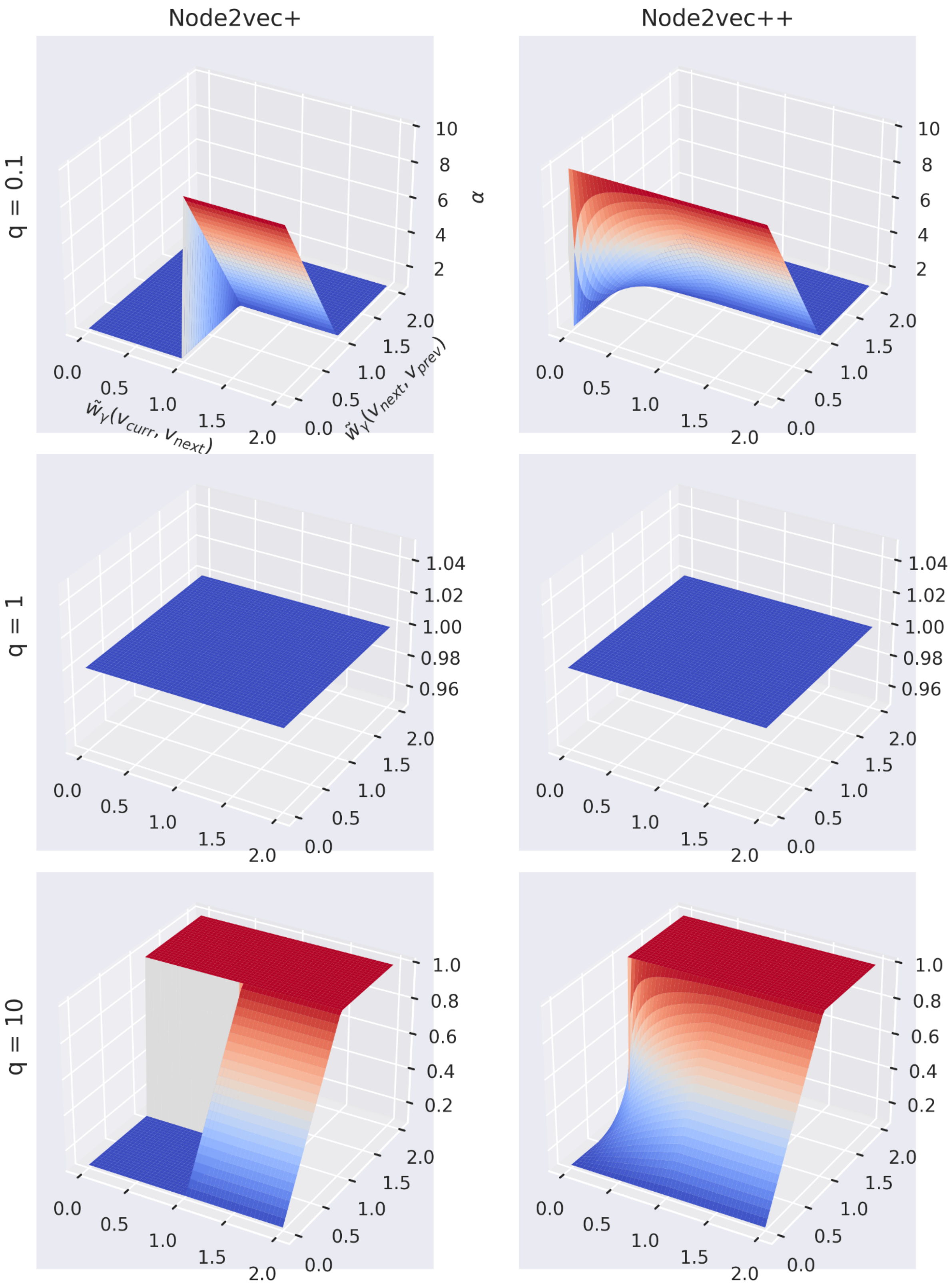

Figure S6. Visualization of the bias factor surface for *node2vec+* and *node2vec++*.

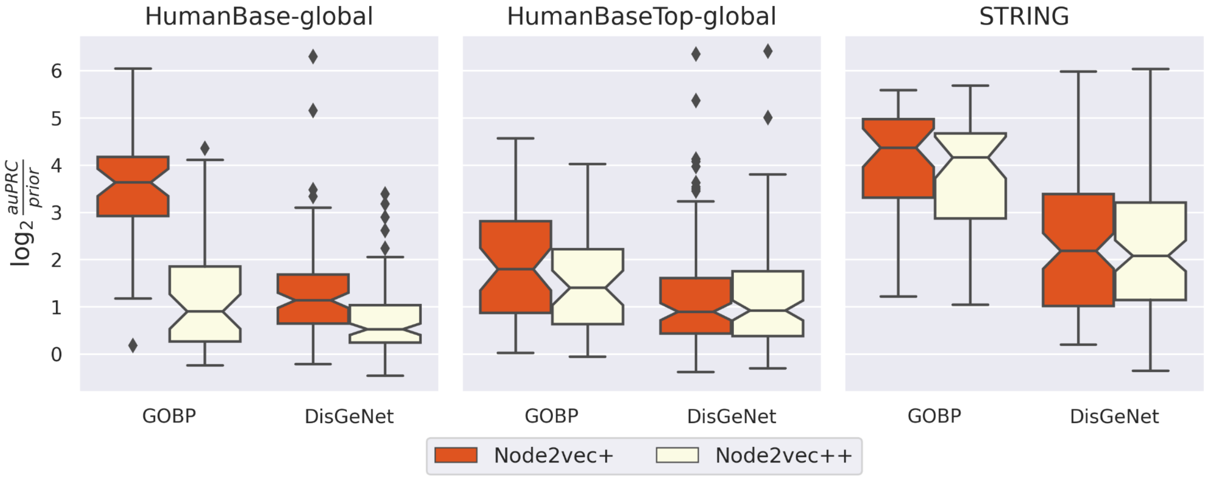

Figure S7. Gene classification task performance comparison between node2vec+ and node2vec++.

Additional results

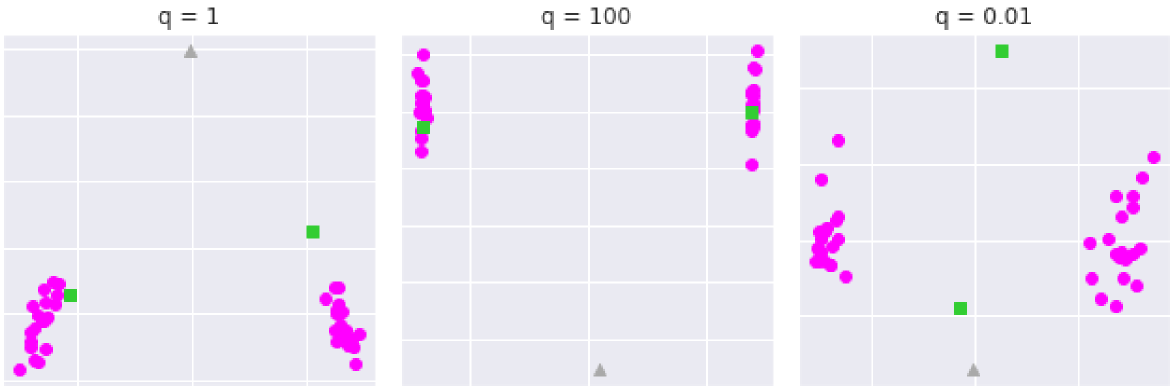

Figure S8. Clean barbell graph embedding using node2vec+.

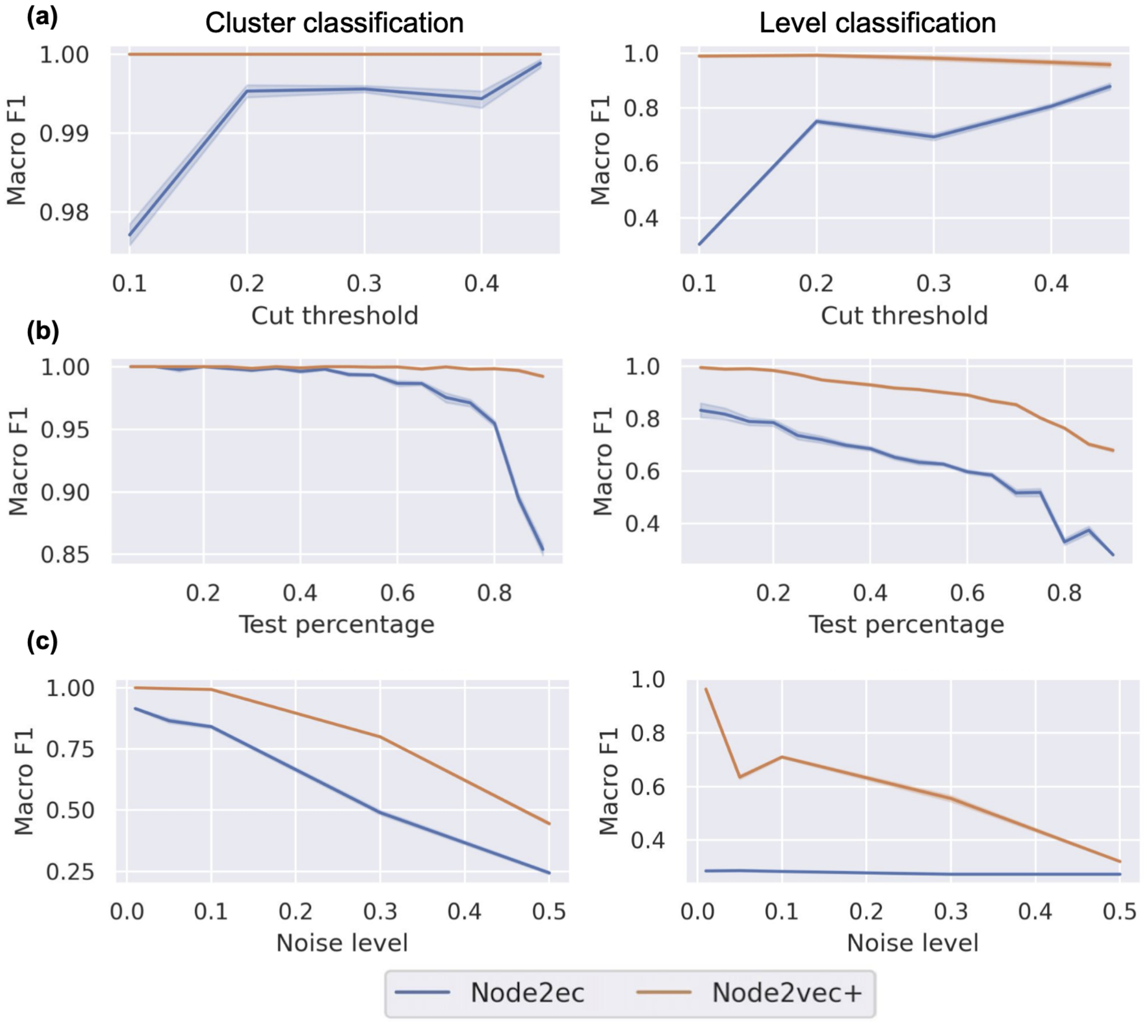

Figure S9. Fine-grained analysis of K3L2. (a) changing sparsification threshold value. (b) changing train/test raio, larger value means less training data. (c) changing noise level during network construction.

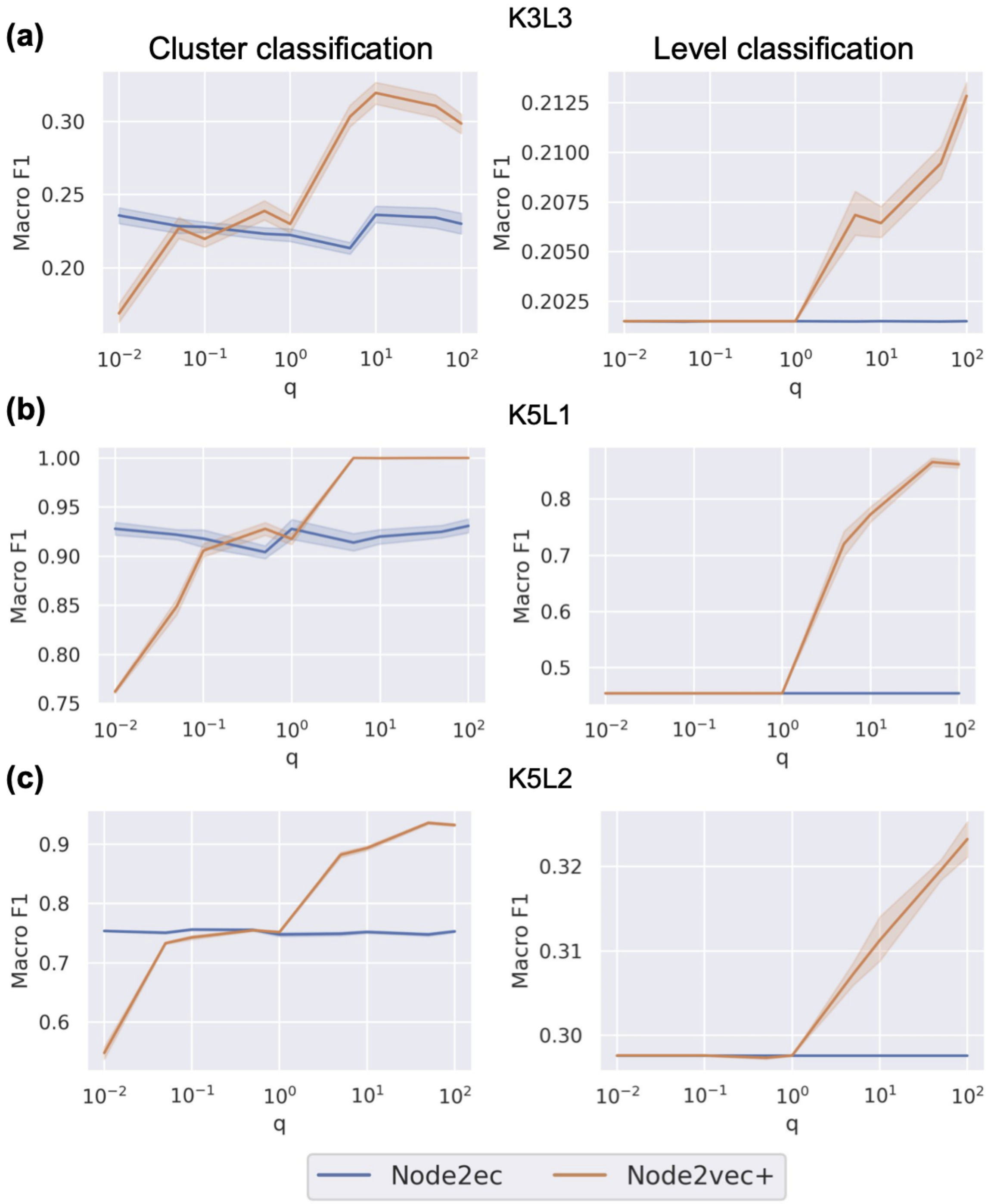

Figure S10. Evaluation of other hierarchical cluster graphs.

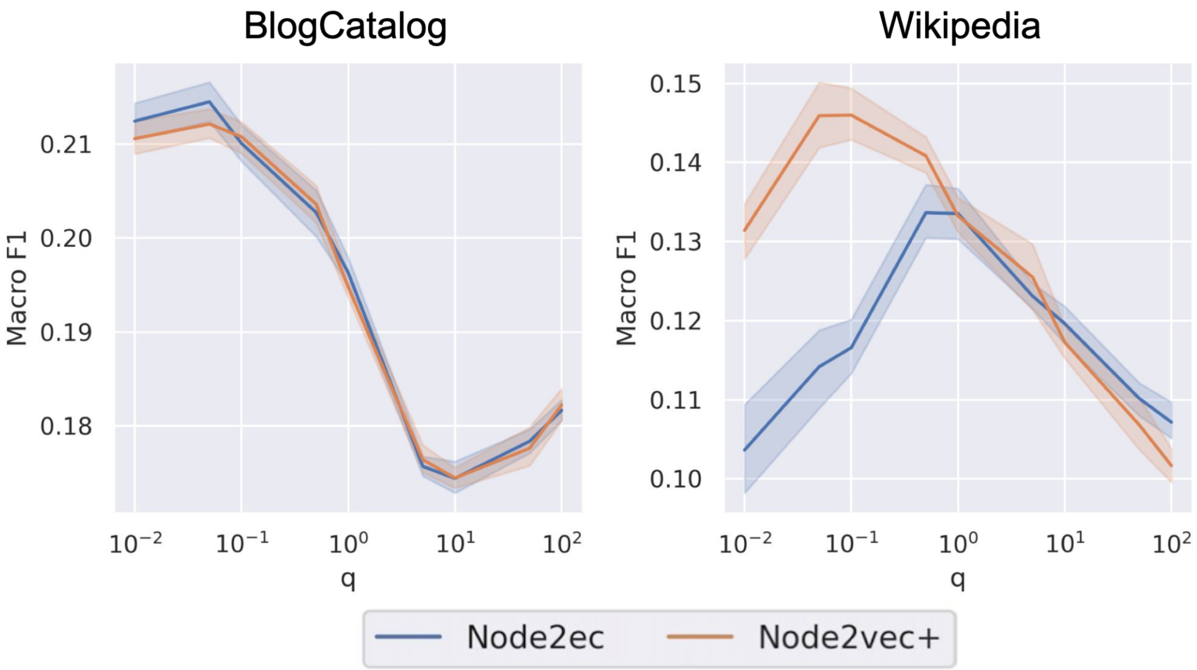

Figure S11. Multi-label classification benchmarks using BlogCatalog and Wikipedia. Data are obtained from https://snap.stanford.edu/node2vec

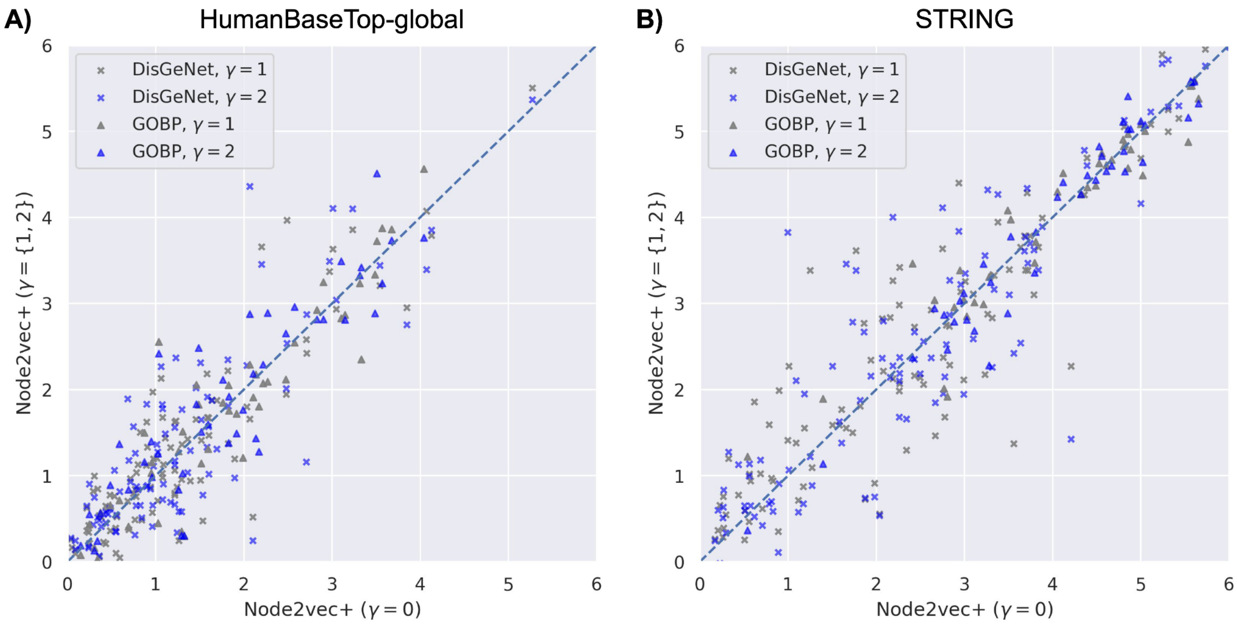

Figure S12. Effects of gamma on HumanBaseTop-global and STRING

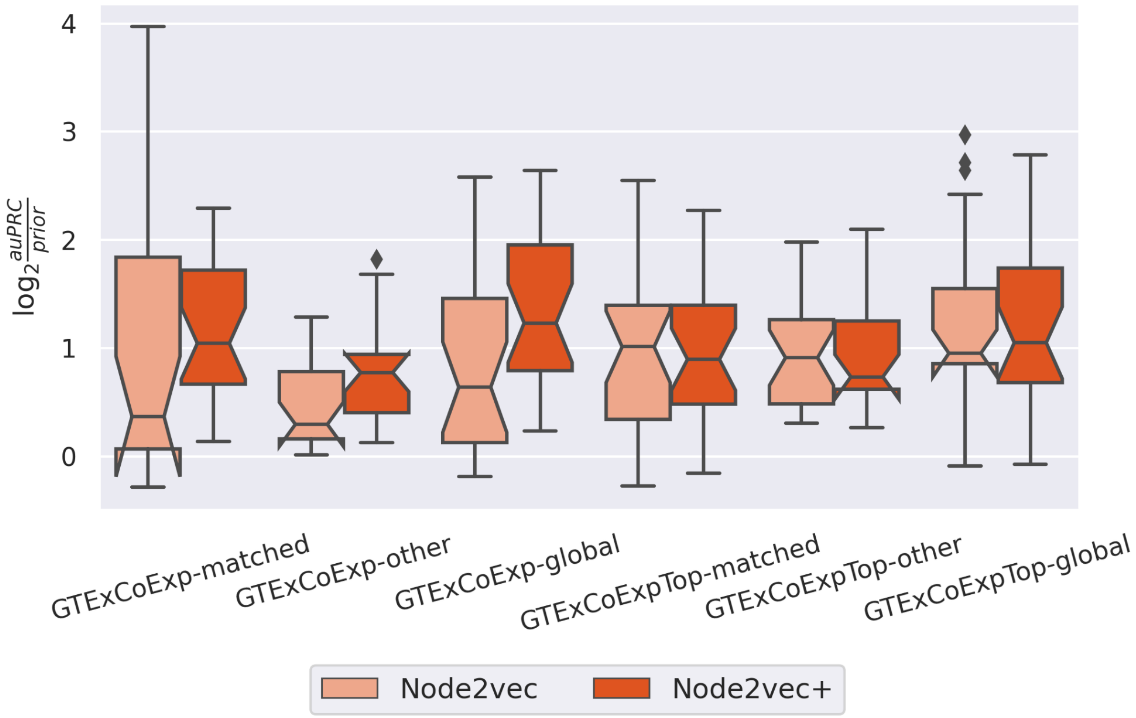

Figure S10. Tissue-specific functional gene classification tasks using GTExCoExp.
